# Mitochondrial membrane potential: a trait involved in organelle inheritance?

**DOI:** 10.1098/rsbl.2015.0732

**Published:** 2015-10

**Authors:** Liliana Milani

**Affiliations:** Department of Biological, Geological and Environmental Sciences, University of Bologna, Bologna, Italy

**Keywords:** mitochondrial inner membrane potential, doubly uniparental inheritance, strictly maternal inheritance, microtubule transport, preformation, epigenesis

## Abstract

Which mitochondria are inherited across generations? Are transmitted mitochondria functionally silenced to preserve the integrity of their genetic information, or rather are those mitochondria with the highest levels of function (as indicated by membrane potential Δ*ψ*m) preferentially transmitted? Based on observations of the unusual system of doubly uniparental inheritance of mitochondria and of the common strictly maternal inheritance mode, I formulate a general hypothesis to explain which mitochondria reach the primordial germ cells (PGCs), and how this happens. Several studies indicate that mitochondrial movements are driven by microtubules and that mitochondria with high Δ*ψ*m are preferentially transported. This can be applied also to the mitochondria that eventually populate embryonic PGCs, so I propose that Δ*ψ*m may be a trait that allows for the preferential transmission of the most active (and healthy) mitochondria. The topics discussed here are fundamental in cell biology and genetics but remain controversial and a subject of heated debate; I propose an explanation for how a Δ*ψ*m-dependent mechanism can cause the observed differences in mitochondrial transmission.

## 1. Mitochondrial inheritance

There is an ongoing debate about which mitochondria are transmitted to progeny and inherited across generations. Some authors postulate that transmitted mitochondria are inactive, whereas others think that the more active mitochondria are preferentially inherited. Interesting theories based on observations in several animal species arise from both viewpoints [1–3]. The main rationale of the ‘inactive theory’ derives from recognition of the need for a functional, undamaged mitochondrial genome, protected from deleterious mutations caused by oxidative damage induced by mitochondrial activity [1,2]. By contrast, the ‘active theory’ affirms that mitochondrial activity is an indication of correct functioning, so mechanisms that favour the inheritance of highly active mitochondria would be adaptive [3].

How can the delivery of functional mitochondria to the germ line, and thus to the next generation, be assured? Several studies indicate that mitochondrial movements are driven by microtubules. In *Drosophila*, for example, the localization of mitochondria around the fusome, the cytoplasmic structure that allows material transport to the oocyte, is dependent on microtubules [4]. Some fusome-associated mitochondria are transported to the oocyte and form an evolutionarily conserved structure, the Balbiani body (Bb), which supplies mitochondria to the germ cells of the next generation. This aggregation of organelles and molecules has been observed in the developing oocytes of a large number of animals, and the selective transport of highly functional mitochondria to the Bb is thought to result in preferential transmission of a subset of mitochondria to the germ line [5–7].

In zebrafish, a selective accumulation of mitochondria with high inner membrane potential (Δ*ψ*m) in the Bb was recently documented [6,7], supporting the theory that highly active mitochondria are preferentially imported into the germ line. The Bb appears to be a dynamic structure, with mitochondria coming and leaving according to their Δ*ψ*m [6]. The presence of high Δ*ψ*m would indicate the integrity of mtDNA, and mitochondria with such a phenotype would have a better chance to attach to microtubules, be recruited into the Bb, and be preferentially carried into embryonic primordial germ cells (PGCs); by contrast, mitochondria with defective functioning would be excluded [7].

The control of ion gradients across the mitochondrial inner membrane is central to bioenergetics, and mitochondria have evolved a variety of mechanisms to move ions and metabolites across their inner membrane [8]. Δ*ψ*m is not only determined by the activity of gene products involved in oxidative phosphorylation (encoded by the nucleus or by the mitochondrion) but is also influenced by the cellular metabolic status and various physiological cues. Thus, even though low Δ*ψ*m is not directly indicative of genetic defects in mitochondria, high Δ*ψ*m is an indication of a functional genotype.

## 2. Sperm mitochondria

The mitochondrial lineage carried by the spermatozoon is commonly not inherited. For this reason, not much interest has been raised about the consequences of mitochondrial activity in spermatozoa, which, considering their role in fertilization, must be particularly active (with high ATP production and consumption). Sperm mitochondria have high Δ*ψ*m not only in species with strictly maternal inheritance of mitochondria (SMI), but also in species in which mitochondria carried by spermatozoa are transmitted to the next generation [3]. Observations on a bivalve mollusc species showing doubly uniparental inheritance of mitochondria (DUI), in which mitochondria are naturally transmitted through males, show that spermatozoon mitochondria do have high Δ*ψ*m [3]. In female embryos of species with DUI, mitochondria from the spermatozoa are excluded from the germ line and transmission is similar to that in SMI organisms; conversely, in male embryos, mitochondria from the eggs are excluded from the germ line. This mitochondrial inheritance through males is thought to be allowed by a mechanism that leads spermatozoon mitochondria to escape degradation in male embryos. A model was proposed and progressively revised to explain how such a mechanism functions [9]. According to this model, the transmission of sperm mitochondria is controlled by three nuclear genes: W, X and Z. Factor W labels sperm mitochondria during spermatogenesis and, upon fertilization, factor X, expressed in the egg, recognizes W causing sperm mitochondria degradation in a mechanism that could be similar to (if not the same as) that observed in some mammals [10]. Factor Z (unique to DUI species) interferes with X–W interaction, allowing sperm mitochondria to reach germ cells in males. Specific nuclear sex-biased genes were proposed to be part of this mechanism [10], together with factors of mitochondrial origin; novel mitochondrial genes with a putative viral origin could also explain the acquired capability of mitochondria from the fertilizing spermatozoon to avoid degradation and invade the germ line in male embryos [11].

Given that sperm-derived mitochondria segregate in the germ line of DUI males, they have to find a way to colonize PGCs. In male embryos, they start their migration by being aggregated in proximity to the first cleavage furrow, whereas in female embryos they are dispersed. The midbody, a cytoplasmic structure formed by the compression of the microtubule spindle during the first embryonic cleavage, was proposed to be involved in the positioning of spermatozoon mitochondria in the region that eventually gives rise to germ cells [12].

## 3. How are mitochondrial activity and segregation linked?

As recently reported in neurons, mitochondrial trafficking is tightly associated with the ability of mitochondria to produce ATP and be highly functional. As observed in Parkinson's disease, mitochondrial dysfunctions can lead to a deficient ATP supply to microtubule motor proteins, leading to the disruption of mitochondrial axonal transport [13]. It is noteworthy that molecules which increase mitochondrial Δ*ψ*m also enhance mitochondrial transport. The majority of long distance mitochondrial transport in higher eukaryotic cells is via motor proteins moving mitochondria along microtubules [14]: mitochondria supply the energy for motor proteins for their transport along the cytoskeleton tracks toward areas with high energy demands. The capacity of mitochondria to produce ATP is, therefore, fundamental for their transfer, and mitochondrial arrest and degradation have been shown to be owing to interrupted ATP production and Δ*ψ*m loss [14].

Sperm mitochondria have to be particularly efficient in ATP production, especially in animals with external fertilization such as bivalves, and this could be a preadaptation enabling preferential transport by carriers through the microtubule network.

The above-mentioned data together suggest a way for DUI male-transmitted mtDNA to colonize the male germ line: spermatozoon mitochondria, having escaped degradation, may be preferentially carried into PGCs by microtubules because of their high Δ*ψ*m. This mechanism would easily explain the ‘active’ behaviour (and movements) of sperm mitochondria in male embryos of DUI species [12]. It is possible that some egg-derived mitochondria with high Δ*ψ*m are also segregated into male embryo PGCs; in this case, additional mechanisms would act allowing only germ cells containing spermatozoon-derived mitochondria to differentiate into male gametes [15].

What happens to sperm mitochondria in embryos of SMI species, in which they are not transmitted to the progeny? To ensure SMI, and homoplasmy, diverse mechanisms appear to act in different organisms and these mechanisms can involve either an active degradation of sperm mitochondria in the embryo (or even sperm mtDNA elimination before fertilization) or an early segregation to specific blastomeres. Because sperm mitochondria in SMI zygotes are also likely to be those with the highest Δ*ψ*m, they can be preferentially transported and segregated to specific blastomeres. Indeed, this appears to occur when mechanisms for their degradation fail or are delayed (e.g. [16]).

## 4. Germ line specification and mitochondrial segregation in germ cells

**Figure 1.**
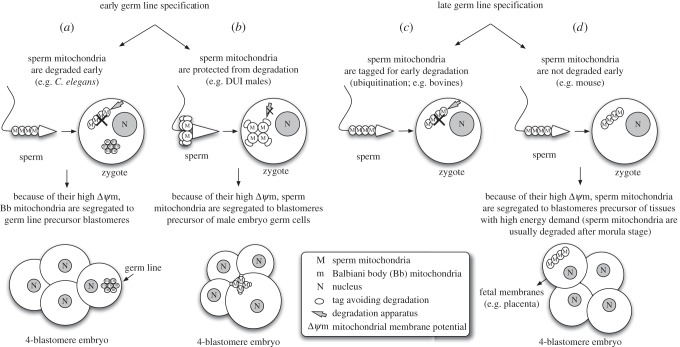
Germ line specification and mitochondrial segregation in germ cells. The timing of germ line specification would enable different mitochondrial segregation outcomes, as highlighted in different organisms. (*a*,*b*) In animals with an early mechanism of germ line specification, the hypothesized segregation of the most active mitochondria in gonadic presumptive blastomeres has to take place contextually. Examples: (*a*) in *C. elegans*, sperm mitochondria are degraded early by autophagy. This allows the segregation in germ line precursors of egg mitochondria with the highest inner membrane potential (Δ*ψ*m). A similar process is predicted for DUI females. (*b*) In DUI males, the degradation of sperm mitochondria is prevented, and, owing to their having the highest Δ*ψ*m, they are segregated to the blastomere precursor of male embryo germ cells. (*c*,*d*) When germ line specification is driven by inductive signals from surrounding tissues at a later developmental stage (as in mammals), at least two pathways of sperm mitochondria elimination are possible. Examples: (*c*) in some mammal species, such as bovines, sperm mitochondria are ubiquitinated and degraded shortly after fertilization owing to degradation tags attached during spermatogenesis or spermiation. (*d*) In the mouse, sperm mitochondria degradation appears to be postponed, but early segregation of these mitochondria into a specific blastomere actually prevents their spread. Therefore, mitochondria carried by the spermatozoon would have been already degraded (ubiquitination; *c*) or isolated in separate tissues (fetal membranes; *d*) when a subset of mitochondria is segregated into germ cell precursors.

Based on what is observed in the unusual system of DUI and in animals with SMI [3–7,10–15], I propose a general hypothesis to explain how mitochondria reach PGCs. In order to be generalizable, such a hypothesis needs to take into account the different mechanisms of germ line specification, and the moment in which mitochondria and other material are set aside to be inherited through the germ line must be considered. Germ line determination can be achieved through the action of inherited material (preformation) or inductive signals (epigenesis) [17]. In the case of preformation (e.g. *Caenorhabditis elegans*), maternal products accumulate in a specific region of the egg or early embryo, and cells acquiring that material begin to differentiate into germ cells [17]. By contrast, in epigenesis there is no predefined localized determinant: instead, cells in the embryo rely on positional information and intercellular communication to become committed to germ cell fate. This occurs later in development, and PGCs are, therefore, established in a more advanced developmental stage (e.g. mouse) [17]. In animals with an early mechanism of germ line specification, the material determining the cell fate is sequestered early on in specific blastomeres, and for this reason the segregation of the most active mitochondria in gonadic presumptive blastomeres has to take place contextually (e.g. *C. elegans* and bivalves; figure 1*a,b*). When germ line specification happens at a later stage of development, owing to inductive signals from surrounding tissues (as in mammals), at least two modes of elimination of sperm mitochondria are possible. In some species, such as bovines [18], sperm mitochondria are ubiquitinated and thus degraded shortly after fertilization (figure 1*c*). In the mouse, the degradation of sperm mitochondria appears to be postponed; nevertheless, their early segregation in one specific blastomere (that for fetal membranes) before the four-cell stage actually prevents their spread in different tissues, thus avoiding heteroplasmy [16] (figure 1*d*). Therefore, spermatozoon mitochondria would have been already degraded (as a result of ubiquitin tagging, e.g. bovines) or isolated in separate tissues (e.g. fetal membranes in mouse) when a subset of mitochondria is segregated into germ cell precursors.

In summary, Δ*ψ*m can be a simple and effective system allowing the most active mitochondria to reach specific locations. Of course, genomic background and timing of action (in the presence of either early or late mechanisms of germ line specification) can influence the outcome of the segregation mechanism. The process, based on Δ*ψ*m, can result in the segregation of spermatozoon mitochondria into the germ line in DUI male embryos [3,9–12]. Δ*ψ*m could also allow particularly active egg mitochondria to end up in the Bb during oogenesis [4–7]. This is what would happen in SMI species and in DUI females because of degradation or inactivation of sperm mitochondria preventing their transport along microtubules, allowing the recruitment of egg mitochondria only. In mice, mitochondria with high Δ*ψ*m might be segregated in fetal membranes as an adaptive consequence of the high energy demand for nutrient transport typical of embryonic annexes, and the same process could be extended to spermatozoon mitochondria, contextually assuring their exclusion from the animal body.

In any case, to better understand the mechanisms of mitochondrial inheritance it will be essential to analyse deeply the relationship between mitochondrial activity, Δ*ψ*m, and Δ*ψ*m-dependent trafficking mechanisms. Interestingly, sperm mtDNA has occasionally been detected in ovarian tissue of newborn mice [16]. This may suggest that paternal mtDNA transmission to the progeny through germ cells might occur in species with SMI, if the mechanisms of inactivation/degradation of sperm mitochondria do not act properly. Analyses uncoupling these degradation mechanisms will be useful to answer important open questions about mitochondrial inheritance. Despite the differences observed among various taxa, mitochondrial inheritance may be controlled in general by the same shared mechanism.
